# Feasibility of subtotal esophagectomy with systematic lymphadenectomy in selected elderly patients with esophageal cancer; a propensity score matching analysis

**DOI:** 10.1186/s12893-019-0617-2

**Published:** 2019-10-15

**Authors:** Mitsuro Kanda, Masahiko Koike, Chie Tanaka, Daisuke Kobayashi, Masamichi Hayashi, Suguru Yamada, Goro Nakayama, Kenji Omae, Yasuhiro Kodera

**Affiliations:** 1 Department of Gastroenterological Surgery (Surgery II), Nagoya University Graduate School of Medicine, 65 Tsurumai-cho, Showa-ku, Nagoya, 466-8550 Japan; 2 Department of Innovative Research and Education for Clinicians and Trainees (DiRECT), Fukushima Medical University Hospital, 1 Hikariga-oka, Fukushima, 960-1295 Japan

**Keywords:** Esophageal cancer, Elderly, Subtotal esophagectomy, Safety, Prognosis

## Abstract

**Background:**

The global increase in elderly populations is accompanied by an increasing number of candidates for esophagectomy. Here we aimed to determine the postoperative outcomes after subtotal esophagectomy in elderly patients with esophageal cancer.

**Methods:**

Patients (*n* = 432) with who underwent curative-intent transthoracic subtotal esophagectomy with 2- or 3-field lymphadenectomies for thoracic esophageal cancer were classified as follows: non-elderly (age < 75 years, *n* = 373) and elderly (age ≥ 75 years, *n* = 59) and groups. To balance the essential variables including neoadjuvant treatment and stage of progression, we conducted propensity score analysis, and clinical characteristics, perioperative course and prognosis were compared.

**Results:**

After two-to-one propensity score matching, 100 and 50 patients were classified in the non-elderly and elderly groups. The elderly group had more comorbidities and lower preoperative cholinesterase activities and prognostic nutrition indexes. Although incidences of postoperative pneumonia, arrhythmia and delirium were slightly increased in the elderly group, no significant differences were observed in overall incidence of postoperative complications, rates of repeat surgery and death caused by surgery, and length of postoperative hospital stay between the two groups. There were no significant differences in disease-free and disease-specific survival as well as overall survival between the two groups.

**Conclusion:**

Older age (≥75 years) had limited impact on morbidity, disease recurrence, and survival after subtotal esophagectomy. Therefore, age should not prevent older patients from benefitting from surgery.

## Background

Esophageal cancer is the sixth on the list of cancers with the highest mortality rates [1]. Moreover, the increasing general life expectancy leads to a larger number of elderly patients diagnosed as esophageal cancer [2]. Although esophagectomy is still positioned as the cornerstone treatment for esophageal cancer, it is associated with high morbidity and mortality rates for elderly patients despite recent advances in the surgical practice [1, 3, 4]. Moreover, there is controversy about whether long-term outcomes after esophagectomy in elderly patients are worse compared to those in younger patients [5, 6].

Physiological changes with advancing age lead to a decline in physiological reserve that potentially places elderly patients at greater risks of adverse events during early postoperative courses after esophagectomy [7]. Moreover, particularly in elderly patients, dysfunction of vital organs such as the heart, lungs or kidneys is associated frequently with esophageal cancer [6, 8]. Surgeons are typically more reluctant to perform esophagectomy for elderly patients due to the aggressiveness of surgery and high incidence of organ insufficiency.

Therefore, we aimed to compare perioperative characteristics and prognosis of elderly patients with esophageal cancer to those of non-elderly patients.

## Methods

### Ethics approval and consent to participate

All procedures performed were in accordance with the ethical standards of the responsible committee on human experimentation (institutional and national) and with the Helsinki Declaration of 1964 and later versions. This study was approved by the Institutional Review Board of Nagoya University (approval number 2017–0475) and written informed consent for surgery and usage of clinical data was obtained from all participants.

### Selection of patients

**Fig. 1:**
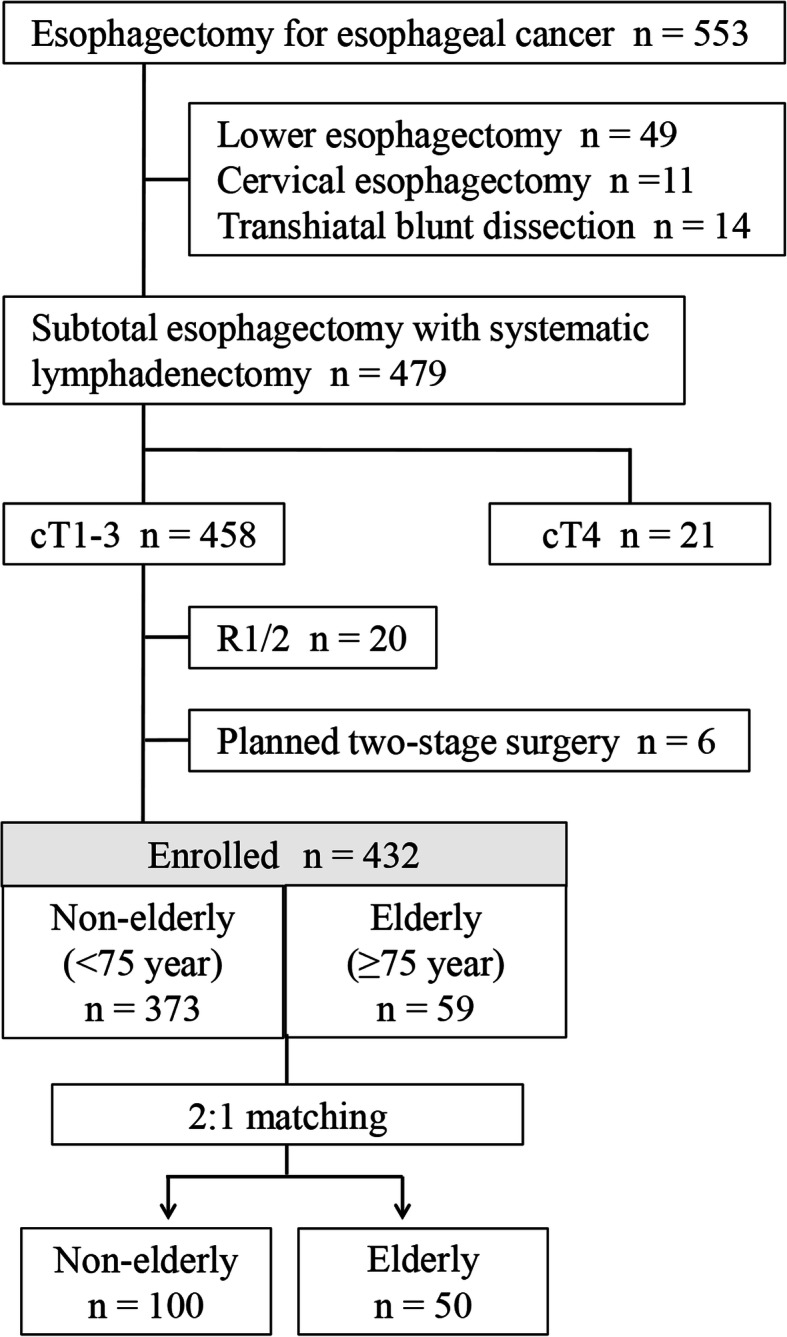
Study design

The study flow-chart is shown in Fig. 1. Consecutive patients (*n* = 553) underwent esophagectomy for esophageal cancer at the Nagoya University Hospital (Department of Gastroenterological Surgery) between February 2005 and March 2017. We retrieved data for 432 patients in accordance with the criteria as follows: subtotal esophagectomy with systematic 2- or 3-field lymphadenectomy; clinical T1–3 esophageal cancer according to the Union for International Cancer Control (UICC) Classification (8th Edition); and R0 resection [9]. Patients who underwent planned two-stage surgery were excluded. Patients were classified into the non-elderly (age < 75 years, *n* = 373) or elderly (age ≥ 75 years, *n* = 59) groups. We used the propensity score matching to balance in essential variables for the comparison analyses that followed. Propensity scores were estimated using a logistic regression model based on sex, neoadjuvant treatment, operative approaches (open or thoracoscopic surgery), number of field dissected, tumor size, pathological T factor, N factor and tumor nodes metastasis (TNM) stage. Two-to-one matching without replacement was performed, and the resulting score-matched pairs were used in subsequent analyses.

### Patient management

The medical team cooperatively managed patients’ physical condition and comorbidities before surgery. No preoperative intervention (e.g. nutritional support and rehabilitation) was routinely provided specifically for elderly patients. All patients underwent subtotal esophagectomy with systematic 2- or 3-field lymphadenectomy. This procedure comprised subtotal esophageal resection through a right thoracotomy or thoracoscopic approaches and a 2- or 3-field lymphadenectomy of nodes at the cervical, mediastinal and intra-abdominal area [10]. The reconstruction method and route were determined according to the patient’s condition and the surgeon’s discretion. Based on evidence from the JCOG9907 study, neoadjuvant treatment is performed in patients with clinical stage II-IV esophageal cancer [11]. We consider postoperative adjuvant chemotherapy (mainly fluorouracil plus cisplatin) for patients who met following criteria; i) pathological stage II-IV, ii) no neoadjuvant treatment, iii) tolerability of chemotherapy and iv) patient consent.

A routine postoperative follow-up screening including a physical, analyses of blood chemistry and tumor markers (squamous cell carcinoma [SCC] antigen and carcinoembryonic antigen) was provided every 3 months for the first and second year and every six months thereafter. Enhanced computed tomography (chest and abdominal cavity) once every 6 months and yearly thereafter. If the patient had a specific symptom, examinations were conducted sooner as needed.

### Comparisons between groups

Preoperative background data included demographics, performance status by the Eastern Cooperative Oncology Group classification, comorbidities, physical condition, blood test results, prognostic nutritional index (PNI = 10 × serum albumin [g/dl] + 0.005 × total lymphocyte count [/mm^3^]), and Controlling Nutritional Status (CONUT) score [12, 13]. The TNM Classification of Malignant Tumors, 8th Edition was used to determine pathological stage [9]. A postoperative short-term outcome was evaluated based on 90-day postoperative mortality, a morbidity rate, and duration of postoperative hospitalization. We employed the Clavien-Dindo classification as a comprehensive evaluation method for postoperative complications [14]. To compare long-term outcomes, disease-free and disease-specific survival as well as overall survival were analyzed.

### Statistical analysis

To compare the two groups, we used a qualitative χ^2^ and quantitative Mann–Whitney’s test. Survival curves were drawn using the Kaplan–Meier method. Survival differences were assessed using the Cox proportional hazards model. For all statistical analysis, JMP 13 software (SAS Institute Inc., NC, USA) was used. *P* < 0.05 represents a statistically significant difference.

## Results

### Patients’ backgrounds

**Fig. 2:**
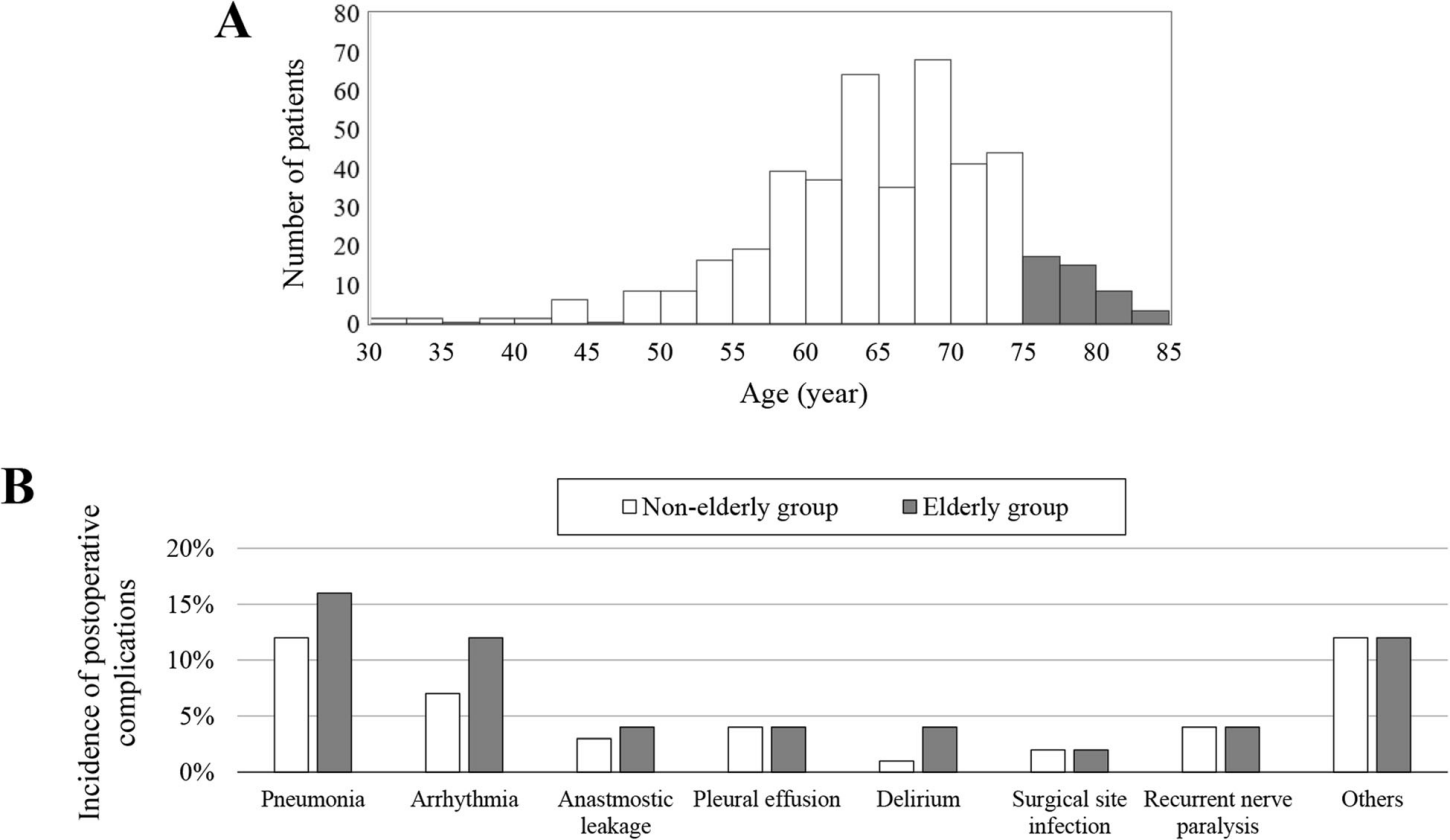
(**a**) Age distribution of patients who underwent subtotal esophagectomy. (**b**) Incidence of postoperative complications according to age

Age distribution of the 432 patients was presented in Fig. 2a. The mean age was 65.8 ± 8.3 (standard deviation, SD) years, and the female:male ratio was 5.4:1. The median follow-up duration was 45.3 months. Before propensity score matching, 373 and 59 patients were classified in the non-elderly and elderly groups, respectively. As shown in Table 1, there were significant differences between the non-elderly and elderly groups in neoadjuvant treatment, number of field dissected, tumor size and pathological TNM stage. After two-to-one propensity score matching, 100 and 50 patients were classified in the non-elderly and elderly groups. Neoadjuvant treatment, number of field dissected, tumor size and pathological TNM stage were balanced by propensity score matching (Table 1).

**Table 1:** Patient characteristics before and after propensity score matching

Characteristic	Unmatched comparison			Matched comparison		
	Non-elderly group (*n* = 373)	Elderly group (*n* = 59)	Standardized difference	Non-elderly group (*n* = 100)	Elderly group (*n* = 50)	Standardized difference
Sex (male/female)	314 / 59	59 / 9	0.016	84 / 16	42 / 8	0.000
Neoadjuvant treatment						
Not performed	149 (40%)	34 (58%)	0.359	53 (53%)	29 (58%)	0.101
Performed	224 (60%)	25 (42%)		47 (47%)	21 (42%)	
Surgical approach						
Open	346 (93%)	52 (88%)	0.158	89 (89%)	45 (90%)	0.033
Thoracoscopic	27 (7%)	7 (12%)		11 (11%)	5 (10%)	
Number of field dissected						
2-field dissection	221 (59%)	49 (83%)	0.528	78 (78%)	40 (80%)	0.049
3-field dissection	152 (41%)	10 (17%)		22 (22%)	10 (20%)	
Tumor size (mm), mean ± SD	35.4 ± 21.6	45.2 ± 23.6	− 0.432	38.3 ± 21.0	44.1 ± 21.6	−0.212
T factor						
pT0/pTis	25 (7%)	2 (3%)	0.351	1 (1%)	0	0.159
pT1	157 (42%)	29 (49%)		51 (51%)	27 (54%)	
pT2	54 (14%)	7 (12%)		11 (11%)	6 (12%)	
pT3	129 (35%)	21 (36%)		37 (37%)	17 (34%)	
pT4	8 (2%)	0		0	0	
N factor						
pN0	172 (46%)	30 (51%)	0.242	45 (45%)	25 (50%)	0.183
pN1	102 (27%)	17 (29%)		30 (30%)	15 (30%)	
pN2	65 (18%)	10 (17%)		20 (20%)	9 (18%)	
pN3	34 (9%)	2 (3%)		5 (5%)	1 (2%)	
TNM stage						
0	17 (4%)	2 (3%)	0.556	1 (1%)	0	0.3000
I	97 (26%)	23 (39%)		36 (36%)	21 (42%)	
II	108 (29%)	10 (17%)		20 (20%)	9 (18%)	
III	103 (28%)	22 (38%)		36 (36%)	19 (38%)	
IV	48 (13%)	2 (3%)		7 (7%)	1 (2%)	

*SD*, standard deviation

The elderly group was more likely to have significantly more overall comorbidities and cardiovascular disease (Table 2). The preoperative levels of serum cholinesterase were significantly lower in the elderly group (Table 2). The elderly group had significantly lower preoperative PNI values. No significant differences were found between CONUT scores (Table 2).

**Table 2:** Patients’ demographics and preoperative clinical characteristics

Characteristic	Non-elderly group (*n* = 100)	Elderly group (*n* = 50)	*P*
Age (years), mean ± SD	65.0 ± 6.9	77.2 ± 2.1	< 0.001
Sex (male/female)	84 / 16	42 / 8	1.000
Performance status (0/1)	99 / 1	50 / 0	0.367
Comorbidity (%)			
Any^a^	28 (28%)	23 (46%)	0.030
Cardiovascular disease^a^	10 (10%)	12 (24%)	0.026
Respiratory disease	3 (3%)	4 (8%)	0.186
Cerebrovascular disease	7 (7%)	4 (8%)	0.823
Renal dysfunction	1 (1%)	2 (4%)	0.234
Diabetes mellitus	11 (11%)	8 (16%)	0.393
History of cancer (%)	16 (16%)	7 (14%)	0.747
Brinkman index ≥1000 (%)	31 (31%)	17 (34%)	0.711
Excessive alcohol consumption	57 (57%)	26 (52%)	0.562
Body mass index, mean ± SD	21.0 ± 3.1	21.1 ± 2.8	0.721
Blood test, median (range)			
Total lymphocyte count (/mm^3^)	1600 (500–3400)	1450 (500–2900)	0.071
Albumin (g/dl)	4.1 (3.0–5.0)	3.9 (2.9–4.9)	0.092
Cholinesterase (IU/l)	276 (117–462)	248 (96–369)	0.015
Cholesterol (mg/dl)	188 (101–360)	184 (118–261)	0.579
Creatinine (mg/dl)	0.8 (0.2–1.6)	0.9 (0.5–1.6)	0.077
CEA (ng/ml)	2.5 (0.4–9.5)	2.5 (0.9–20.1)	0.987
SCC (ng/ml)	1.2 (0.3–7.9)	1.3 (0.5–5.4)	0.371
PNI, median (range)	49.5 (34.5–60.0)	46.3 (35.0–57.5)	0.019
CONUT score, median (range)	1 (0–5)	1 (0–5)	0.733
Neoadjuvant treatment (%)	47 (47%)	21 (42%)	0.562

*SD* standard deviation, *CEA* carcinoembryonic antigen, *SCC* squamous cell carcinoma antigen, *PNI* prognostic nutritional index, *CONUT* Controlling Nutritional Status. ^a^Hypertension is not included

### Intraoperative findings and pathological data

The data described in this section are shown in Table 3. Surgical procedure was evenly balanced by the propensity score matching between the two groups. SCC was a dominant histopathologic type and accounted for 94 and 98% of patients in the non-elderly and elderly groups. The elderly group had marginally greater amount of intraoperative blood loss and larger frequency of intraoperative transfusion, but smaller number of dissected lymph nodes, though there were no statistically significant differences.

**Table 3:** Intraoperative and pathological characteristics

Characteristic	Non-elderly group (*n* = 100)	Elderly group (*n* = 50)	*P*
Surgical approach			
Open	89 (89%)	45 (90%)	0.851
Thoracoscopic	11 (11%)	5 (10%)	
Number of field dissected			
2-field dissection	78 (78%)	40 (80%)	0.777
3-field dissection	22 (22%)	10 (20%)	
Reconstruction			
Jejunal flap	22 (22%)	10 (20%)	0.777
Gastric tube	78 (78%)	40 (80%)	
Operative time (minutes), mean ± SD	473 ± 111	475 ± 122	0.889
Intraoperative blood loss (ml), median (range)	439 (31–1959)	517 (187–1886)	0.051
Intraoperative transfusion (%)	13 (13%)	13 (26%)	0.053
Number of dissected lymph nodes mean ± SD	43.7 ± 14.8	40.1 ± 15.8	0.097
Histopathologic Type (%)			
Squamous cell carcinoma	94	49	0.192
Adenocarcinoma	2	1	
Others	4	0	
Tumor size (mm), mean ± SD	38.3 ± 21.0	44.1 ± 21.6	0.141
Multiple lesion (%)	15 (15%)	8 (16%)	0.873
T factor			
pT0/pTis	1 (1%)	0	0.806
pT1	51 (51%)	27 (54%)	
pT2	11 (11%)	6 (12%)	
pT3	37 (37%)	17 (34%)	
pT4	0	0	
N factor			
pN0	45 (45%)	25 (50%)	0.779
pN1	30 (30%)	15 (30%)	
pN2	20 (20%)	9 (18%)	
pN3	5 (5%)	1 (2%)	
TNM stage			
0	1 (1%)	0	0.546
I	36 (36%)	21 (42%)	
II	20 (20%)	9 (18%)	
III	36 (36%)	19 (38%)	
IV	7 (7%)	1 (2%)	

SD, standard deviation

### Postoperative short-term outcomes

Postoperative complications of at least grade 2 were experienced by 36 (36%) and 22 (44%) patients in the non-elderly and elderly groups (Table 4). Moreover, 12 (12%) and 4 (8%) patients in the non-elderly and elderly groups experienced postoperative complications of at least grade 3 according to the Clavien-Dindo classification. Although incidences of postoperative pneumonia, arrhythmia and delirium were slightly increased in the elderly group (Fig. 2b), there were no significant differences in overall incidence of postoperative complications (at least grade 2), rates of repeat surgery and operative death between the two groups (Table 4). The mean lengths of postoperative hospitalizations were similar between groups. The elderly group was less likely to receive postoperative adjuvant treatment (Table 4).

**Table 4:** Postoperative course

Characteristic	Non-elderly group (*n* = 100)	Elderly group (*n* = 50)	*P*
Postoperative complications (%)			
CD grade 2 or more	36 (36%)	22 (44%)	0.345
CD grade 3 or more	12 (12%)	4 (8%)	0.445
Reoperation (%)	6 (6%)	2 (4%)	0.599
Operative death^a^ (%)	0	1 (2%)	0.137
Length of postoperative stay (days), mean ± SD	28.1 ± 20.5	26.0 ± 20.0	0.395
Postoperative adjuvant therapy (%)	12 (12%)	1 (2%)	0.022

*CD* Clavien-Dindo classification, *SD* standard deviation. ^a^Death within 90 days after surgery

### Long-term outcomes

**Fig. 3:**
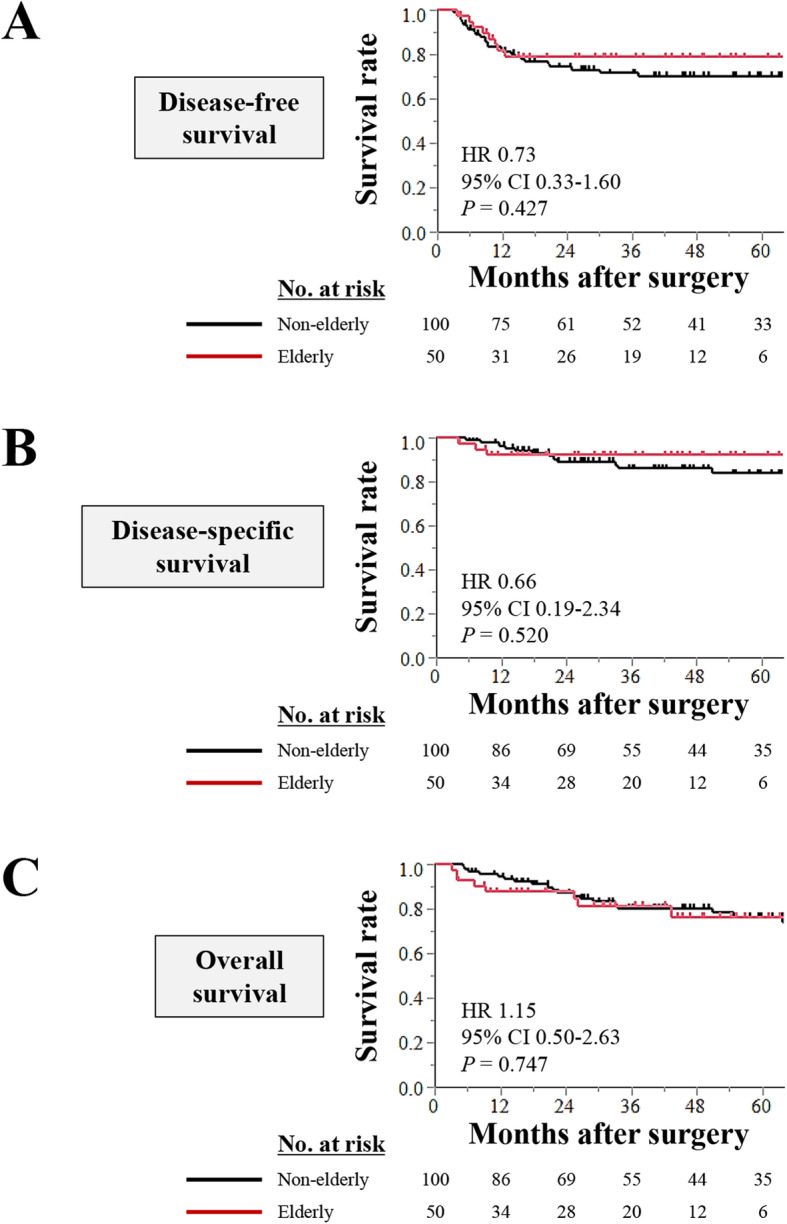
Prognosis of patients who underwent a curative resection for esophageal cancer according to age. (**a**) Disease-free, (**b**) disease-specific and (**c**) overall survival

No significant differences were observed in the curves for disease-free and disease-specific survival of the two groups (Fig. 3 a and b). Moreover, overall survival rates were comparable between the two groups (Fig. 3c).

## Discussion

Here we sought to determine the outcomes of elderly patients with esophageal cancer after subtotal esophagectomy for. After adjustment by propensity score matching, we found that no significant differences were observed in overall incidence of postoperative complications, the rates of repeat surgery and death during surgery, length of postoperative hospitalization, and prognosis between the non-elderly and elderly patients.

Aging is a poor descriptor of physical, mental, or medical functional condition [15, 16]. Accordingly, selecting appropriate treatment in elderly patients with esophageal cancer is always challenging because impaired functional and nutritional status, comorbidities, cognitive function, quality of life after surgery, and life expectancy should be concerned [17, 18]. From lack of an established definition, the definitions of elderly are arbitrary and differ among studies (e.g., > 65, > 70, or > 80 years). Here we defined 75 years as “elderly,” because it is considered as the late-stage of elderly by Japanese society.

Our comparison here of the elderly and non-elderly groups reveal that the rate of repeat surgery, mortality and length of postoperative hospitalization, despite a slightly higher incidence of pneumonia, arrhythmia and delirium, and more frequent disadvantages including overall comorbidity, cardiovascular disease as well as impaired immune-nutritional status in the former group. These findings indicate that subtotal esophagectomy is feasible for selected elderly patients when the medical team provides an appropriate perioperative care. The possible explanations of acceptable outcomes of elderly patients are as follows: 1) The multidisciplinary medical team comprised surgeons, anesthesiologists, geriatrics physicians, physical therapist, and a nutritionist who supported patients. 2) Patients at high risk of adverse cardiopulmonary events underwent planned two-stage surgery or were subjected to different treatment. 3) Patients’ postoperative complications were intensively managed to prevent exacerbation of their disease. Despite acceptable short-term outcomes, the length of hospital stay was long (mean, 28.1 and 26.0 days in the non-elderly and elderly groups). In Japan, the length of hospital stay tends to be much longer than that in the Western countries because of the difference in social and medical systems [2, 5, 7, 8, 19]. Patients typically return home to daily life directly after discharge so that they stay at the hospital until they get substantial recovery, fair oral intake, and removal of all drainage tubes.

Correlations between age and prognosis of patients with esophageal cancer is a subject of debate. Diversity in definitions of elderly patients, the inclusion criteria, methods of analysis likely contribute to the discrepancy [7, 8, 18–20]. In the present study, long-term outcomes in the elderly group was comparable to those of the non-elderly group after adjustment of essential variables including neoadjuvant treatment, number of field dissected, tumor size and TNM stage with the propensity score matching. Further prospective studies addressing survival, cost-effectiveness, and postoperative quality of life will be required to establish treatment guidelines for elderly esophageal cancer patients.

This present study has limitations, such as a limited number of patients and potential selection biases due to the retrospective nature even after propensity score matching. In the present study, only patients who underwent subtotal esophagectomy were analyzed. Frail patients who had severe comorbidities or poor performance status were considered to be unfit for esophagectomy and subjected to different treatments. The lack of objective assessment criteria for postoperative quality of life prevented us from deepening the discussion. Unfortunately, survival data of patients undergoing palliative care that possibly support our conclusion are unavailable this time.

## Conclusion

Short-term and long-term outcomes after subtotal esophagectomy were comparable between the elderly and non-elderly patients. Our findings indicate that subtotal esophagectomy is justified for selected elderly esophageal cancer patients and should not be withheld because of a patients’ age.

## Data Availability

The datasets generated and analyzed during the current study are available from the corresponding author on reasonable request.

## Abbreviations

CONUT: Controlling Nutritional Status
PNI: Prognostic nutritional index
SCC: Squamous cell carcinoma
SD: Standard deviation
TNM: Tumor nodes metastasis
UICC: Union for International Cancer Control
